# Development of semicircular canal occlusion

**DOI:** 10.3389/fnins.2022.977323

**Published:** 2022-08-19

**Authors:** Su Fei, Li Guangfei, Meng Jie, Gao Yiling, Cai Mingjing, Zhang Qingxiang, Meng Wei, He Shuangba

**Affiliations:** ^1^Department of Otorhinolaryngology Head and Neck Surgery, School of Medicine, Nanjing Tongren Hospital, Southeast University, Nanjing, China; ^2^Department of Pharmacy, School of Medicine, Nanjing Tongren Hospital, Southeast University, Nanjing, China

**Keywords:** benign paroxysmal positional vertigo, Meniere's disease, labyrinthine fistula, semicircular canal occlusion, superior semicircular canal dehiscence syndrome

## Abstract

Surgical treatment of vertigo is performed with in-depth study of inner ear diseases. Achieving an effective control of vertigo symptoms while reducing damage to hearing and reducing surgical complications is the principle followed by scholars studying surgical modalities. Semicircular canal occlusion is aimed at treatment of partial peripheral vertigo disease and has attracted the attention of scholars because of the above advantages. This article provides a review of the origins of semicircular canal occlusion, related basic research, clinical applications, and the effects of surgery on vestibular and hearing function.

## Introduction

Vertigo is a challenging and common disease entity. For a long time, no advancement in the diagnosis and treatment has been observed, and guidelines for treatment and diagnosis are not standardized, leading to inaccuracies. Medical treatment is often administered as a symptomatic treatment of vertebrobasilar insufficiency in clinics. In recent years, research on vertigo has made an important breakthrough, which has become the focus of multi-disciplinary attention. Presently, the direction of development of diagnosis and treatment technology is standardized and specialized. This is manifested in the diagnosis, treatment, daily management, and rehabilitation of vertigo. Clinicians pay close attention to the surgical treatment of especially some vertigo disorders.

Through the study of an animal model and the evaluation of postoperative effect of a large number of clinical cases, indications, perioperative treatment, surgery matters needing attention, postoperative vestibular rehabilitation, and follow-up of semicircular canal occlusion have gradually been standardized. The postoperative effect is also gradually emerging. This paper summarizes relevant studies by experts to facilitate clinical application.

## Origin of semicircular canal occlusion

Semicircular canal occlusion was first initiated by Parnes and McClure (1990) in 1990 to treat benign paroxysmal positional vertigo (BPPV). The first routine treatment for vertigo is comprehensive drug therapy, with the main purpose of regulating autonomic nerve function and improving inner ear microcirculation. However, for recurrent and increasing intractable vertigo, clinicians consider surgical intervention, with unilateral semicircular canal occlusion becoming a research hotspot due to the decreased risk of trauma and good hearing protection; furthermore, the curative effect has been affirmed.

Minor et al. (1998) and Minor (2000) first reported findings related to symptomatic superior semicircular canal fistula in 1998. Five cases of surgical treatment *via* the middle cranial fossa approach through packing or surface covering the semicircular canal fistula have been reported. Brantberg et al. (2001) promoted filling of the superior semicircular canal through the mastoid approach to avoid the complications of craniotomy and temporal lobe injury. However, the transmastoid superior semicircular canal occlusion was widely criticized when it was first used because semicircular canal fistula is not fully demonstrated, and labyrinthine opening can easily lead to sensorineural hearing loss, tinnitus, and postoperative vertigo. In addition, the material and technology of semicircular canal occlusion also limited the application of this technology.

However, a number of subsequent studies have confirmed that in the case of BPPV or superior semicircular canal fistula, transmastoid semicircular canal occlusion can effectively relieve vertigo and preserve hearing. Thereafter, some scholars explored the application of triple semicircular canal occlusion (TSCO) in the treatment of Meniere's disease. Gentine et al. (2008) reported that 11 patients with Meniere's disease were treated with horizontal semicircular canal occlusion, and nine cases of vertigo were controlled. Yin et al. (2008) reported that TSCO was used to treat three patients with recurrent vertigo after endolymphatic sac decompression or drainage. Vertigo was controlled effectively.

## Basic research on semicircular canal occlusion

The animal model of semicircular canal occlusion has been used to study vestibular function compensation. Unilateral labyrinthectomy in albino rats and TSCO in guinea pigs are recognized as vestibular compensatory animal models. As the largest vestibular nucleus, some electrophysiological data show that the medial vestibular nucleus may be one of the important areas of vestibular nerve compensation after semicircular canal occlusion. One study (Zhang et al., 2015) used guinea pigs to establish a model for unilateral horizontal semicircular canal occlusion to simulate the clinical semicircular canal occlusion operation. Then, the level of serotonin in medial vestibular nucleus was monitored by *in vivo* microdialysis combined with high- performance liquid chromatography and electrochemical detection. It was found that unilateral horizontal semicircular canal occlusion could increase the level of serotonin in the medial vestibular nucleus. It is suggested that serotonin and central compensation may play an important role in the compensatory process of residual vestibular function. Concurrently, the animal model of vestibular compensation with unilateral horizontal semicircular canal was successfully established.

Yin et al. (2006) verified the effectiveness and safety of TSCO in the ear with endolymphatic hydrops. They used the endolymphatic sac closure method to establish endolymphatic hydrops model in 20 guinea pigs. Among them, 12 animals underwent TSCO 120 days after operation, and eight were sacrificed for morphological observation to determine the existence of an endolymphatic fluid. The auditory and vestibular functions of the subjects were detected prior to the occurrence of endolymphatic hydrops to 1 month after TSCO, and the success of endolymphatic hydrops and semicircular canal occlusion was confirmed by morphological observation. Finally, it was concluded that TSCO effectively eliminated the response of semicircular canals to rotation and caloric test. It was safe in the ear with endolymphatic hydrops, and simultaneously, the static compensation for imbalance was fast and complete. It was suggested that TSCO was also an effective method to control rotational vertigo in ears with endolymphatic hydrops.

## Application of semicircular canal occlusion in treatment of BPPV

BPPV, also known as otolithic vertigo, is an idiopathic paroxysmal vestibular organ disease with nystagmus and transient paroxysmal vertigo caused by head position change. It is self-limited and the most common form of peripheral vestibular vertigo (Bhattacharyya et al., 2017). Adler (1897) first described this disease in 1897. In 1921, Bárány (1920) reported a patient with sudden vertigo nystagmus in the right recumbent position. The symptoms disappeared after 30 s. Barany believes that this phenomenon is caused by damage to otolith. In 1952, Dix and Hallpike (1952) introduced the term BPPV, expounded the disease systematically, and put forward the gold standard Dix-Hallpike test for the diagnosis of the disease. With the deepening of scholars' understanding of the disease, different treatments have emerged. Treatment methods are otolith manual reduction, otolith machine reduction, vestibular rehabilitation training, and surgery. Among them, for patients with refractory BPPV after manual reduction, surgical treatment can be used.

Currently, surgical treatment for BPPV mainly includes posterior ampullary neurotomy and semicircular canal occlusion (Zhang and Fan, 2014; Kalmanson et al., 2021). The former was first proposed by Gacek (1974) and Gacek (1984) to treat BPPV by cutting off the posterior ampullary nerve that innervates the posterior semicircular canal. The operation may lead to serious complications such as sensorineural deafness, cerebrospinal fluid leakage, and facial paralysis. Posterior semicircular canal occlusion was first proposed by Parnes and McClure (1990). Its purpose is to make the crest physiologically fixed by closing the liquid space between the crest top and the site of obstruction. Presently, a posterior semicircular canal occlusion is most widely used in the treatment of BPPV, and its curative effect has been affirmed.

Walsh et al. (1999) performed posterior semicircular canal occlusion in 13 patients with refractory BPPV, with special emphasis on their long-term follow-up. The mean follow-up was 66 months (range, 29–119 months). All patients reported complete and immediate resolution of their positional vertigo, which has been maintained in the long term. Most patients, however, reported some postoperative transient unsteadiness which lasted up to 4 weeks. All patients developed a transient mild conductive hearing loss postoperatively, which usually resolved within 4 weeks. Five patients developed a transient mild sensorineural hearing loss at high frequency which resolved in all cases within 6 months. There were no reports of sensorineural hearing loss nor tinnitus in the long term. Parnes (1996) reported that positional vertigo was completely relieved after posterior semicircular canal occlusion, and most patients had temporary mixed hearing loss after surgery, and their hearing later returned to the preoperative level. The efficacy of this surgical method in the treatment of refractory BPPV has been affirmed; temporary vertigo or hearing loss may occur after surgery and gradually disappear after surgery (Uetsuka et al., 2012).

## Application of semicircular canal occlusion in the treatment of Meniere's disease

Meniere's disease is a clinical condition defined by spontaneous vertigo attacks (each lasting 20 min to 12 h) with documented low to midfrequency sensorineural hearing loss in the affected ear before, during or after one of the episodes of vertigo. It also presents with fluctuating aural symptoms (hearing loss, tinnitus, or ear fullness) in the affected ear (Basura et al., 2020). Its incidence varies from 3.5 to 513 per 100,000 persons (Zhang et al., 2019) and is most common between the ages of 40 and 60 years (Basura et al., 2020).

Although Meniere's disease is caused by a variety of factors, such as microcirculatory disorders, viral infections, allergies, autoimmune reactions, and genetic factors, most scholars believe that the pathological basis of Meniere's disease is that when the inner ear endolymph accumulates, vertigo occurs when the potassium-rich endolymph leaks into sodium-rich perilymph. In 1938, Hallpike and Cairns (1938) confirmed the role of endolymphatic hydrops in Meniere's disease through the study of animal models. Although there is currently no cure, more than 85% of patients with Meniere's disease are helped by either changes in lifestyle and medical treatment, or minimally invasive surgical procedures such as intratympanic steroid therapy, intratympanic gentamicin therapy, and endolymphatic sac surgery (Sajjadi and Paparella, 2008). In addition, the surgical treatment of Meniere's disease is a research hotspot in recent years.

The surgical treatment of Meniere's disease has a history of more than 100 years, with Frazier first transecting the eighth cerebral nerve through the posterior fossa approach to treat Meniere's disease in 1908. The traditional surgical treatment of Meniere's disease mainly includes endolymphatic sac surgery, vestibular neurotomy, and labyrinthectomy. In recent years, progress has been made in the surgical treatment of Meniere's disease, such as the application of semicircular canal occlusion, development of endolymphatic aqueduct obstruction, clinical application of vestibular implantation, and application of cochlear implant with labyrinthine surgery (Cowan et al., 2020).

Surgical treatment includes endolymphatic sac surgery, TSCO, vestibular neurotomy, and labyrinthectomy, etc (Editorial Board of Chinese Journal of Otorhinolaryngology Head Neck Surgery; Society of Otorhinolaryngology Head Neck Surgery Chinese Medical Association, 2017). The indications are patients with frequent and severe vertigo and ineffective conservative treatment for more than 6 months. And the semicircular canal occlusion is a new surgical treatment. The short-term control rate of vertigo was 100% and the long-term control rate was 95%. It is an ideal surgical treatment, with a very small number of patients having their hearing affected (Jiang et al., 2022). In the Editorial Board of Chinese Journal of Otorhinolaryngology Head Neck Surgery; Society of Otorhinolaryngology Head Neck Surgery Chinese Medical Association (2017), patients with Meniere's disease are divided into four stages, and patients whose hearing level is worse than 70dB are Classified into forth stage. The guideline alse suggests that semicircular canal occlusion is applicable to patients diagnosed stage IV Meniere's disease. Hence, patients with hearing loss above 70 dB should be selected (Editorial Board of Chinese Journal of Otorhinolaryngology Head Neck Surgery; Society of Otorhinolaryngology Head Neck Surgery Chinese Medical Association, 2017), and triple semicircular canals should be blocked concurrently, with the aim to block the movement of lymph in the semicircular canal so as to eliminate vertigo caused by postural changes.

Zhang et al. (2016) conducted semicircular canal occlusion in more than 200 patients with Meniere's disease. The efficacy of semicircular canal occlusion in the treatment of Meniere's disease was studied. The vertigo control rate at short-term follow-up was 100%: 49 patients were followed up for more than 3 years, the total effective rate of vertigo control was 100%, and about 30% of the patients had hearing loss. This shows that semicircular canal occlusion is effective in the treatment of intractable Meniere's disease. The principle is that after the semicircular canal is blocked, the endolymphatic flow is blocked and the rotational vertigo caused by the displacement of the ampullary crest is eliminated. Although the application of this procedure is limited in some patients with a low cranial plate or mastoid sclerosis, semicircular canal occlusion may still be the first choice for most patients with intractable Meniere's disease (with severe hearing loss).

## Application of semicircular canal occlusion in treatment of labyrinthine fistula caused by middle ear cholesteatoma

Labyrinthine fistula is a complication of otitis media, with a reported incidence ranging from 4 to 15% (Ostri and Bak-Pedersen, 1989; Dornhoffer and Milewski, 1995; Copeland and Buchman, 2003; Stephenson and Saliba, 2011; Prasad et al., 2013). It is mostly caused by cholesteatoma invading the labyrinthine bone and can also be observed in ulcerative otitis media and mastoiditis, occasionally after middle ear mastoid surgery or trauma. Due to the damage of the bone wall connected to the cochlea, the endosteum is in direct contact with the cholesteatoma and causes damage to the endosteum and results in perilymph overflowing to the mastoid cavity of the middle ear; labyrinthine fistula can not only cause vertigo (Villari et al., 2021), but also lead to sensorineural hearing loss (Jang and Merchant, 1997). Fistula often occurs in the horizontal semicircular canal and can also be seen in the superior semicircular canal, posterior semicircular canal, and promontory (Bo et al., 2016). The mechanisms leading to bone wall destruction include: (1) localized osteitis caused by chronic infection; (2) non-inflammatory osteolysis mainly caused by cholesteatoma compression and chemicals such as collagenase produced by cholesteatoma matrix. Except for bone resorption, there are common pathological changes, such as fibrous tissue hyperplasia and new bone formation in the fistula (Ostri and Bak-Pedersen, 1989).

At present, there are many classification criteria for labyrinthine fistula, including Dornhofer's (1995) fistula, which are widely used at present and can be divided into three types (Dornhoffer and Milewski, 1995):

Type I: The labyrinthine bone is absorbed but the membranous labyrinth is intact.
Type II: The bone labyrinth and membranous labyrinth are destroyed at the same time to form a fistula, and the depth of the fistula is <1 × 2 of the semicircular canal diameter.
Type III: The bone labyrinth and membranous labyrinth are destroyed at the same time to form a fistula. The depth is >1 × 2 of the semicircular canal diameter until amputation.

Quaranta et al. (2009) provides the latest classification, in which labyrinthine fistula can be divided into six stages according to CT and intraoperative findings.

The staging system considers the size and depth of the fistula (Prasad et al., 2013) (see Table 1).

**Table 1:** Fistula staging.

Stage I: Pre-fistula (blue line)
Stage II: Small fistula 2 mm
Stage III: Fistula 2–4 mm
Stage IV: Invasion of one (a) or more (b) semicircular canal/s
Stage V:
(a) Invasion of the vestibule
(b) Invasion of the vestibule and cochlea
Stage VI:
(a) Fistula limited to the stapes footplate
(b) Promontorial fistula

A labyrinthine fistula is usually difficult to diagnose preoperatively. Fistula test and CT of the temporal bone are commonly used to diagnose labyrinthine fistula, but they are not reliable when the test result is negative, and the most reliable method requires careful exploration during surgery. Simultaneously, with the development of diagnostic technology, HIT and semicircular canal multi-planar reconstruction (s-MPR) can be used appropriately to increase the positive rate of preoperative diagnosis. Indeed, the findings of intraoperative exploration is an important basis for diagnosis (D'Albora et al., 2017).

Yamauchi et al. (2014) first reported in 2014 the “underwater” endoscopic labyrinthine fistula closure technique. Thangavelu et al. (2022) reported the effect of “underwater technology” on postoperative hearing in patients with cholesteatoma complicated with labyrinthine fistula. The “underwater technique” for the removal of cholesteatoma in labyrinthine surgery is a feasible choice to maintain the function of the inner ear and promote the complete removal of cholesteatoma. There is no significant difference in bone conduction threshold before and after the operation. Sensory improvement in 20% of patients was more than 10 dB, and no patients had worsened postoperative sensorineural hearing loss. Some patients had temporary vertigo after the operation, but they eventually recovered. Pace et al. (2022) reported that the hearing of patients with cholesteatoma can be preserved using the “underwater technique” for labyrinthine fistula. Therefore, for patients with destruction of bone labyrinth and membranous labyrinth, it is safe to use the appropriate fascia packing method during operation. Moreover, the operative method is simple, easy, and time-efficient; has fewer complications, and there is no obvious interference to hearing; thus, it is widely applied clinically. Labyrinthine fistula is a common complication of middle ear cholesteatoma; thus, the possibility of labyrinthine fistula should be greatly considered. During the operation, the fistula area should be completely removed and repaired to prevent the disease from worsening (Gersdorff et al., 2000).

## Application of semicircular canal occlusion in treatment of superior semicircular canal dehiscence syndrome

Superior semicircular canal dehiscence syndrome (SCDS) is a vestibular disorder caused by a pathologic third window into the labyrinth, resulted from a bone defect above the superior semicircular canal, which leads to a series of auditory and vestibular function syndromes (Steenerson et al., 2020). Its typical clinical manifestations are vertigo and auditory hypersensitivity caused by strong sound stimulation.

In 1998, Minor et al. (1998) first reported eight cases of SCDS, and since then, many cases of SCDS have been reported (Steenerson et al., 2020; de Wolf et al., 2021; Kontorinis and Gaggini, 2021; Nieto et al., 2021; Ellsperman et al., 2022). The incidence of SCDS is low and its pathogenesis is unknown, and it may be related to head trauma, infection, and pathological changes in the middle ear or adjacent structures (Rosowski et al., 2004; Ward et al., 2017; Mau et al., 2018). Its diagnosis needs to involve clinical manifestations, auditory function examination, vestibular function examination, and imaging examination.

The surgical treatment of SCDS includes the management of the superior semicircular canal and round window, and the operation related to the superior semicircular canal is performed earlier and is more mature. There are relatively few reports of round window surgery, which is a new surgical method, and its long-term effects remain to be observed. Surgical approaches include the middle cranial fossa approach, mastoid approach, external auditory canal approach, and endoscope-assisted surgical approach (Creighton Jr et al., 2021). The above surgical method needs to be realized by different surgical approaches, among which the middle cranial fossa approach and mastoid approach are reported more frequently, the postoperative results are stable, and each has its own advantages (Gersdorff et al., 2022). The external auditory canal approach is often combined with round window surgery, which has the advantage of less trauma. And the modified middle cranial fossa approach combined with ear endoscopy avoids the greater trauma caused by the traditional middle fossa craniotomy and the disadvantage of being unable to observe the dehiscence directly (Tugrul et al., 2022).

Management of superior semicircular canal includes occlusion, capping, and covering. Occlusion of the superior semicircular canal is the most frequently reported operation, which can be achieved by the middle cranial fossa approach and mastoid approach (Allsopp et al., 2020), and there is no difference in the postoperative effect. Semicircular canal occlusion was first reported by Minor et al. (1998). The surgical procedure mainly includes enlarging the dehiscence and filling the lumen of the superior semicircular canal with fascia, bone cement, and other materials. Resurfacing covers the dehiscence of the superior semicircular canal with autogenous tissues such as cartilage and fascia without blocking the superior semicircular canal. Capping is an improvement to the covering technique and uses hydroxyapatite cement instead of its own tissue to cover the dehiscence of the superior semicircular canal; hence, there is no risk of absorption and migration. The success rate of capping is similar to that of occlusion, and it is advantageous in that it cures the Tullio phenomenon and hyperacusis (Vlastarakos et al., 2009; Kaski et al., 2012; Mueller et al., 2014; Smith et al., 2022).

According to the summary of clinical cases, in the superior semicircular canal-related surgery, the postoperative effect of occlusion has been affirmed by scholars worldwide (Hassannia et al., 2019), and the postoperative effects of other surgical methods are still controversial. Superior semicircular canal occlusion is an ideal surgical method for the treatment of superior semicircular canal dehiscence syndrome with a low incidence of complications.

## Effect of semicircular canal occlusion on hearing and vestibular function

The preservation of cochlear function after semicircular canal occlusion is key to its popularization and application (Lin et al., 2021), and experimental studies and clinical applications show that there is a separation system between the superior labyrinth (vestibular labyrinth) and inferior labyrinth (auditory labyrinth), i.e., the membrana limitans and utricle-endolymphatic valve (Smith et al., 2022). The vestibular labyrinth is comprised of the semicircular canals (detecting angular acceleration) and otolith organs (utricle and saccule, which detect linear acceleration and head tilt relative to gravity). Lying just inferior to the utricle is the membranous membrana limitans (ML). Acting as a keystone to vestibular geometry, the ML provides support for the utricular macula and acts as a structural boundary between the superior (pars superior) and inferior (pars inferior) portions of the vestibular labyrinth. The utricle-endolymphatic valve is a living valve-like structure located at the entrance of the utricle in the anterior and inferior walls of the utricle. It is passively closed when the lymphatic pressure in the upper and lower labyrinth changes, which can prevent excessive endolymphatic loss caused by secondary labyrinthine destruction. The relative independence of the internal and external lymphatic system between the superior and inferior labyrinth provides an anatomical basis for semicircular canal occlusion to preserve hearing. In addition, the collapse and adhesion of the membranous semicircular canal caused by inflammation separate the fistula from the adjacent tissue and limit the spread of inflammation to the downward labyrinth. Animal experiments and clinical observations showed that single and multiple semicircular canal occlusion did not affect the function of the cochlea and other vestibular terminal organs other than the blocked semicircular canal.

Stultiens et al. (2021) performed vestibular function examination and hearing test before superior semicircular canal or posterior semicircular canal filling and 1 week, 2 months, and 6 months after the operation. Testing included caloric irrigation test, video Head Impulse Test (vHIT), cervical and ocular Vestibular Evoked Myogenic Potentials (VEMPs) and audiometry. Concurrently, the following oculomotor muscle movement tests were performed to rule out central lesions: smooth pursuit, saccade, optokinetic nystagmus, spontaneous nystagmus, and gaze evoked nystagmus. The results showed that the ipsilateral caloric test decreased in all patients, and the vHIT vestibulo-ocular refexes (VOR) gain decreased in six patients with unilateral semicircular canal. Six months after the operation, the thermal response of six patients recovered to 60% of the preoperative value. The bilateral unblocked semicircular canal vHIT VOR of five of six patients returned to 85% of the preoperative value. Four VEMP reactions in the neck and eyes were preserved in six patients. The results of the hearing function test were similar to those of the vestibular function test; during the 1-week follow-up after the operation, the bone conduction hearing of three of six patients deteriorated, and the 10 dB was even lower than that before the operation. Although one patient sustained a 15- dB injury at 8 Hz, it recovered within 6 months after the operation, and the vestibular function and hearing of patients after semicircular canal occlusion decreased significantly in a short period of time after semicircular canal occlusion.

Yin et al. (2006) concluded that TSCO controls vertigo, is easy to perform, and could be used as an alternative procedure for the treatment of Meniere's disease in selected patients who complain mainly of intractable vertigo. Three patients with Meniere's disease who underwent unsuccessful endolymphatic sac decompression or mastoid shunt was then selected to undergo TSCO.Vertigo control and vestibular and auditory function were measured. The early vestibular symptoms caused by surgery resolved quickly and no hearing deterioration occurred after surgery.

Chen et al. (2010) investigated the safety and efficacy of semicircular canal occlusion for surgical treatment of labyrinthine fistula caused by cholesteatoma. Twenty-two patients with labyrinthine fistula who were treated surgically were enrolled in the study. All patients were treated by completely removing the cholesteatoma matrix followed by semicircular canal occlusion. With a follow-up of at least 6 months, there was no recurrent cholesteatoma in any of the patients. Vertigo disappeared in all the patients. Most patients presented no hearing deteriorate and four of them demonstrated hearing improvement. No patient presented with surgery-related deafness postoperatively.

Although the vestibular function and hearing of most patients recovered continuously during the follow-up period (Gill et al., 2021; Zhang et al., 2021; Kontorinis and Thachil, 2022), some patients still had persistent impairment of their inner ear function. The limitation of this study is that there are few samples, and the changes of biomechanical properties of the inner ear may also include perilymph leakage and postoperative local inflammation caused by tissue injury. This study cannot rule out the influence of related factors. However, with further development of basic scientific research and clinical follow-up monitoring related to semicircular canal occlusion, related research on the effect of semicircular canal occlusion on vestibular function and hearing will be more accurate.

## Summary

**Figure 1:**
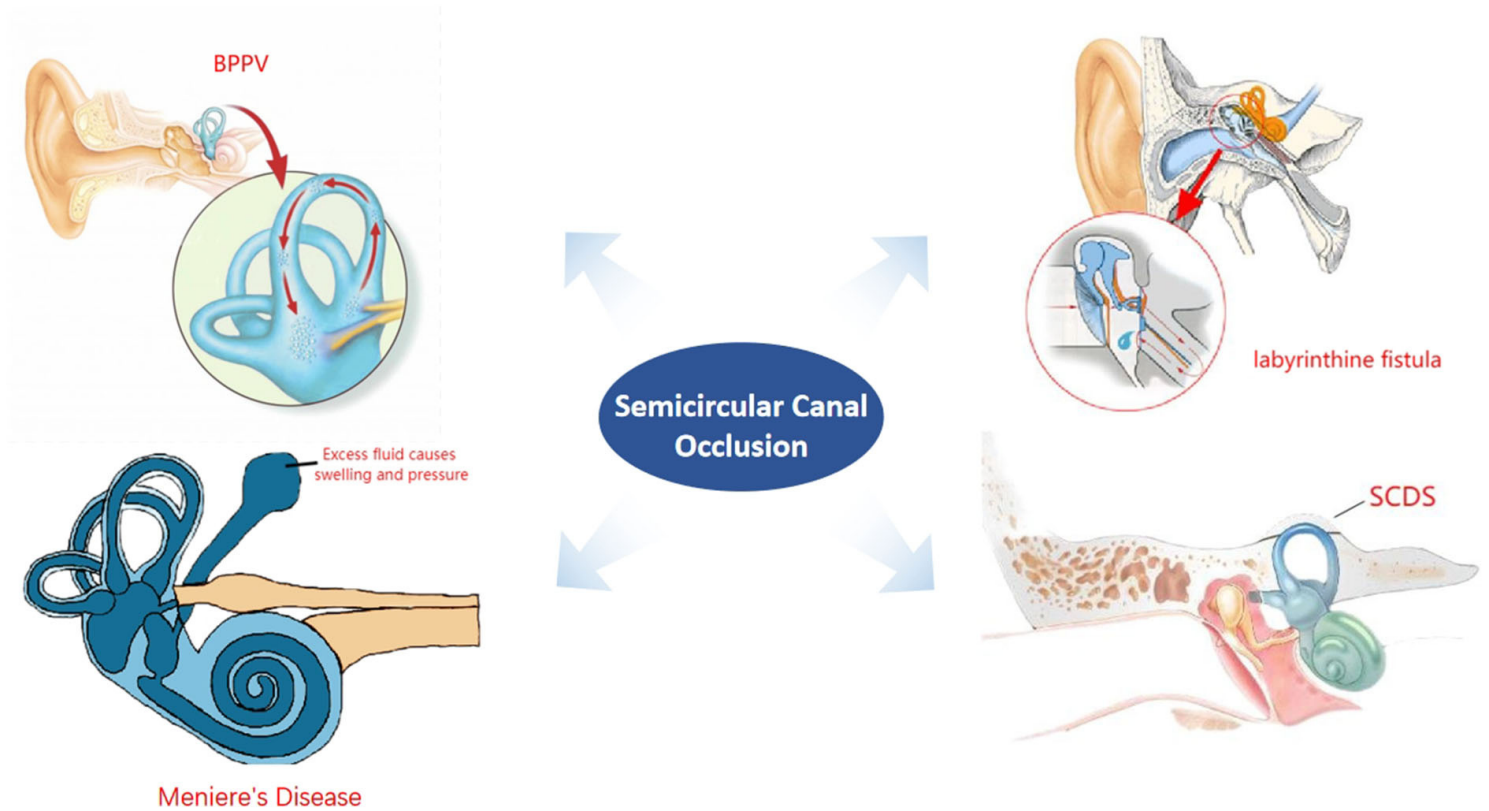
Development of semicircular canal occlusion.

Semicircular canal occlusion is a surgical method that has been highlighted in recent years and has a good effect after surgical treatment of refractory BPPV, intractable Meniere's disease, superior semicircular canal dehiscence syndrome, and middle ear cholesteatoma complicated with labyrinthine fistula. The symptoms of postoperative vertigo can be effectively controlled, the postoperative hearing loss is less, and other serious complications are minimized. These advantages have been recognized by scholars. However, there are still some reports that the hearing of patients after semicircular canal occlusion deteriorates over time (see Figure 1).

The degree of recovery and duration of postoperative vertigo vary from patient to patient, and the exact cause of the decline in inner ear function after semicircular canal surgery is not completely clear; therefore, effective control of the influence of semicircular canal occlusion on vestibular and cochlear organs is an important direction for future research.

## Data availability statement

The original contributions presented in the study are included in the article/supplementary material, further inquiries can be directed to the corresponding authors.

## Author contributions

SF, HS, and MW contributed to study concept. LG contributed to critical revision of the literature. SF and MJ contributed to literature search and drafting of the article. GY contributed to revising english translation and some medical terminology. CM contributed to guiding the submission process. ZQ contributed to English grammar and writing modifications. MW contributed to critical revision. MW and HS contributed to research direction guidance. All authors contributed to the article and approved the submitted version.

## Funding

This work was supported by the National Natural Science Foundation of China (No. 82171153), the Natural Science Foundation of Jiangsu Province (No. BK20211012), Nanjing Medical Science and Technique Development Foundation (No. QRX17033).

## Conflict of interest

The authors declare that the research was conducted in the absence of any commercial or financial relationships that could be construed as a potential conflict of interest.

## Publisher's note

All claims expressed in this article are solely those of the authors and do not necessarily represent those of their affiliated organizations, or those of the publisher, the editors and the reviewers. Any product that may be evaluated in this article, or claim that may be made by its manufacturer, is not guaranteed or endorsed by the publisher.
